# 2-Methylxanthen-9-one

**DOI:** 10.1107/S1600536812007702

**Published:** 2012-02-29

**Authors:** N. Vinutha, Sumati Anthal, V. Lakshmi Ranganatha, Shaukath Ara Khanum, D. Revannasiddaiah, Rajni Kant, Vivek K. Gupta

**Affiliations:** a Department of Studies in Physics, University of Mysore, Mysore 570 006, India; b Post-Graduate Department of Physics and Electronics, University of Jammu, Jammu Tawi 180 006, India; c Department of Chemistry Yuvaraja’s College, University of Mysore, Mysore 570 005, India

## Abstract

In the title compound, C_14_H_10_O_2_, the tricycle is not planar, being bent with a dihedral angle of 4.7 (1)° between the two benzene rings. In the crystal, π–π inter­actions between the six-membered rings of neighbouring mol­ecules [centroid–centroid distances = 3.580 (3) and 3.605 (3) Å] form stacks propagating along [101].

## Related literature

For general background and applications of xanthones, see: Jiang *et al.* (2004 ▶); Sampath & Vijayaraghavan (2007 ▶); Nakatani *et al.* (2002 ▶); Pinto *et al.* (2005 ▶). For related structures, see: Ee *et al.* (2010 ▶); Boonnak *et al.* (2010 ▶). For bond-length data, see: Allen *et al.* (1987 ▶).
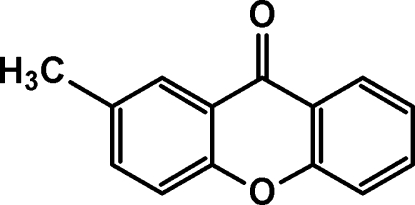


## Experimental

### 

#### Crystal data

C_14_H_10_O_2_
*M* *_r_* = 210.22
Triclinic, 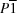

*a* = 8.2678 (7) Å
*b* = 8.5268 (6) Å
*c* = 8.5965 (7) Å
α = 92.650 (6)°
β = 116.592 (8)°
γ = 104.045 (7)°
*V* = 517.28 (7) Å^3^
*Z* = 2
Mo *K*α radiation
μ = 0.09 mm^−1^
*T* = 293 K
0.30 × 0.20 × 0.20 mm

#### Data collection

Oxford Diffraction Xcalibur Sapphire3 diffractometer
Absorption correction: multi-scan (*CrysAlis PRO*; Oxford Diffraction, 2010 ▶) *T*_min_ = 0.890, *T*_max_ = 1.000
10601 measured reflections
2028 independent reflections
1262 reflections with *I* > 2σ(*I*)
*R*_int_ = 0.033
Standard reflections: ?

#### Refinement

*R*[*F* ^2^ > 2σ(*F* ^2^)] = 0.052
*wR*(*F* ^2^) = 0.152
*S* = 1.04
2028 reflections
146 parameters
H-atom parameters constrained
Δρ_max_ = 0.13 e Å^−3^
Δρ_min_ = −0.15 e Å^−3^

### 

Data collection: *CrysAlis PRO* (Oxford Diffraction, 2010 ▶); cell refinement: *CrysAlis PRO*; data reduction: *CrysAlis PRO*; program(s) used to solve structure: *SHELXS97* (Sheldrick, 2008 ▶); program(s) used to refine structure: *SHELXL97* (Sheldrick, 2008 ▶); molecular graphics: *ORTEP-3* (Farrugia, 1997 ▶); software used to prepare material for publication: *PLATON* (Spek, 2009 ▶).

## Supplementary Material

Crystal structure: contains datablock(s) I, global. DOI: 10.1107/S1600536812007702/cv5249sup1.cif

Structure factors: contains datablock(s) I. DOI: 10.1107/S1600536812007702/cv5249Isup2.hkl

Supplementary material file. DOI: 10.1107/S1600536812007702/cv5249Isup3.cml

Additional supplementary materials:  crystallographic information; 3D view; checkCIF report

## Figures and Tables

**Fig. 1.**
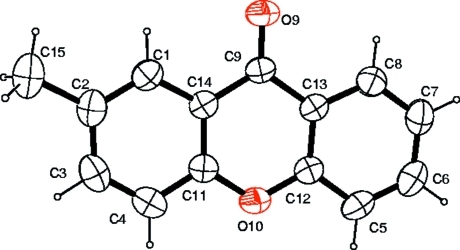
ORTEP view of the molecule with the atom-labeling scheme. The displacement ellipsoids are drawn at the 40% probability level. H atoms are shown as small spheres of arbitrary radii.

## supplementary crystallographic information

### Comment

Xanthones, a particular class of plant phytochemicals from mangosteen, are highly biologically active compounds, which possess anti-inflammatory properties such as COX inhibition, and have cardiovascular protective effects (Jiang *et al.*, 2004; Sampath & Vijayaraghavan, 2007; Nakatani *et al.*, 2002). Many naturally occurring xanthones and their prenylated derivatives are found to exhibit significant biological and pharmacological properties, such as antibacterial, antifungal and anti-tumor activities and it can be inferred that the presence of phenyl groups can be associated with an improvement of potency and selectivity for some of these properties (Pinto *et al.*, 2005). As a large number of biologically active xanthene derivatives with pyran and dihydropyran rings are commonly found in nature, we were interested in obtaining these type of compounds to evaluate their antitumor activity. For this purpose, the title compound, 2-methyl-xanthen-9-one (I), was synthesized.

In (I) (Fig. 1), all bond lengths are within normal ranges (Allen *et al.*, 1987) and comparable to those observed in related structures (Ee *et al.*, 2010; Boonnak *et al.*, 2010). The three ring system is not planar. The dihedral angle between the two benzene rings is 4.7 (1)°. π–π Interactions with distances *Cg*1···*Cg*2^i^ = 3.605 (1) Å (symmetry code: 1 - *x*, -*y*, -*z*); *Cg*2···*Cg*2^i^ = 3.850 (1) Å and *Cg*3···*Cg*1^ii^ = 3.580 (1) Å [symmetry codes: (i) 1 - *x*, -*y*, -*z*; (ii) 2 - *x*, -*y*, 1 - *z*], *Cg*1, *Cg*2 and *Cg*3 are the centroids of C9/C14/C11–C13, C1–C4/C11/C14 and C5–C8/C13/C12 rings, respectively, form stacks of the molecules propagated in [101].

### Experimental

(4-Benzoyl-4-methyl-phenoxy)-acetic acid ethyl ester was achieved by refluxing a mixture of 5.methyl-2-hydroxy benzophenone (2.94 g, 0.013 mol) and ethyl chloroacetate (3.18 g, 0.026 mol) in the presence of dry acetone (50 ml) and anhydrous potassium carbonate (2.69 g, 0.019 mol) for 8 h. The reaction mixture was cooled and solvent was removed by distillation. The residual mass was triturated with cold water to remove potassium carbonate and extracted with ether (3 τimes 50 ml). The ether layer was washed with 10% sodium hydroxide solution (3 τimes 50 ml) followed by water (3τimes30 ml) and then dried over anhydrous sodium sulfate and evaporated to dryness. The crude solid on recrystallization with ethanol afforded (4-benzoyl-4-methyl-phenoxy)-acetic acid ethyl ester with 90% yield. A mixture of (4-benzoyl-4-methyl-phenoxy)-acetic acid ethyl ester (1 g, 0.0033 mol) and sodium hydroxide (0.064 g, 0.0016 mol) in presence of ethyl alcohol (40 ml) was refluxed for about 7–9 hrs. After completion of reaction monitored by TLC, the reaction mixture was cooled and neutralized with 5% sodium carbonate solution. The solvent was removed by distillation and the residual mass was washed with water and recrystallized from methanol to achieve 2-methyl-xanthen-9-one with 70% yield. m.p.369–373 K; IR (Nujol):1665 cm-1 (C=O); 1H NMR (CDCl3): δ 2.3 (s, 3H, Ar—CH3), 6.9–7.6(bm, 7H, Ar—H); Anal. Cal. for C14H10O2 C, 79.98; H, 4.79; Found: C, 79.94; H, 4.76%.

### Refinement

All H atoms were positioned geometrically and were treated as riding on their parent C atoms, with C—H distances of 0.93–0.96 Å; and with *U*_iso_(H) = 1.2-1.5 *U*_eq_(C).

### Crystal data

**Table:**

C_14_H_10_O_2_	*Z* = 2
*M**_r_* = 210.22	*F*(000) = 220
Triclinic, *P*1	*D*_x_ = 1.350 Mg m^−^^3^
Hall symbol: -P 1	Mo *K*α radiation, λ = 0.71073 Å
*a* = 8.2678 (7) Å	Cell parameters from 3639 reflections
*b* = 8.5268 (6) Å	θ = 3.6–29.1°
*c* = 8.5965 (7) Å	µ = 0.09 mm^−^^1^
α = 92.650 (6)°	*T* = 293 K
β = 116.592 (8)°	Block, white
γ = 104.045 (7)°	0.30 × 0.20 × 0.20 mm
*V* = 517.28 (7) Å^3^	

### Data collection

**Table:**

Oxford Diffraction Xcalibur Sapphire3 diffractometer	2028 independent reflections
Radiation source: fine-focus sealed tube	1262 reflections with *I* > 2σ(*I*)
Graphite monochromator	*R*_int_ = 0.033
ω scans	θ_max_ = 26.0°, θ_min_ = 3.6°
Absorption correction: multi-scan (*CrysAlis PRO*; Oxford Diffraction, 2010)	*h* = −10→10
*T*_min_ = 0.890, *T*_max_ = 1.000	*k* = −10→10
10601 measured reflections	*l* = −10→10

### Refinement

**Table:**

Refinement on *F*^2^	Primary atom site location: structure-invariant direct methods
Least-squares matrix: full	Secondary atom site location: difference Fourier map
*R*[*F*^2^ > 2σ(*F*^2^)] = 0.052	Hydrogen site location: inferred from neighbouring sites
*wR*(*F*^2^) = 0.152	H-atom parameters constrained
*S* = 1.04	*w* = 1/[σ^2^(*F*_o_^2^) + (0.0652*P*)^2^ + 0.0822*P*] where *P* = (*F*_o_^2^ + 2*F*_c_^2^)/3
2028 reflections	(Δ/σ)_max_ < 0.001
146 parameters	Δρ_max_ = 0.13 e Å^−^^3^
0 restraints	Δρ_min_ = −0.15 e Å^−^^3^

### Special details

**Table:**

Experimental. *CrysAlis PRO*, Oxford Diffraction Ltd., Version 1.171.34.40 (release 27–08-2010 CrysAlis171. NET) (compiled Aug 27 2010,11:50:40) Empirical absorption correction using spherical harmonics, implemented in SCALE3 ABSPACK scaling algorithm.
Geometry. All e.s.d.'s (except the e.s.d. in the dihedral angle between two l.s. planes) are estimated using the full covariance matrix. The cell e.s.d.'s are taken into account individually in the estimation of e.s.d.'s in distances, angles and torsion angles; correlations between e.s.d.'s in cell parameters are only used when they are defined by crystal symmetry. An approximate (isotropic) treatment of cell e.s.d.'s is used for estimating e.s.d.'s involving l.s. planes.
Refinement. Refinement of *F*^2^ against ALL reflections. The weighted *R*-factor *wR* and goodness of fit *S* are based on *F*^2^, conventional *R*-factors *R* are based on *F*, with *F* set to zero for negative *F*^2^. The threshold expression of *F*^2^ > σ(*F*^2^) is used only for calculating *R*-factors(gt) *etc*. and is not relevant to the choice of reflections for refinement. *R*-factors based on *F*^2^ are statistically about twice as large as those based on *F*, and *R*- factors based on ALL data will be even larger.

### Fractional atomic coordinates and isotropic or equivalent isotropic displacement parameters (Å^2^)

**Table:**

	*x*	*y*	*z*	*U*_iso_*/*U*_eq_	
C1	0.7846 (3)	0.1172 (2)	0.0063 (2)	0.0552 (5)	
H1	0.8299	0.0663	−0.0570	0.066*	
C2	0.7434 (3)	0.2613 (3)	−0.0345 (3)	0.0608 (6)	
C3	0.6748 (3)	0.3344 (3)	0.0625 (3)	0.0672 (6)	
H3	0.6448	0.4315	0.0362	0.081*	
C4	0.6505 (3)	0.2675 (3)	0.1948 (3)	0.0658 (6)	
H4	0.6046	0.3187	0.2574	0.079*	
C5	0.7034 (3)	−0.1210 (3)	0.5685 (3)	0.0623 (6)	
H5	0.6586	−0.0609	0.6249	0.075*	
C6	0.7523 (3)	−0.2566 (3)	0.6273 (3)	0.0705 (7)	
H6	0.7410	−0.2886	0.7248	0.085*	
C7	0.8186 (3)	−0.3476 (3)	0.5441 (3)	0.0707 (7)	
H7	0.8514	−0.4401	0.5855	0.085*	
C8	0.8355 (3)	−0.3005 (3)	0.4002 (3)	0.0601 (6)	
H8	0.8802	−0.3616	0.3445	0.072*	
C9	0.8034 (3)	−0.1098 (2)	0.1823 (2)	0.0487 (5)	
O9	0.8493 (2)	−0.19003 (18)	0.09566 (19)	0.0720 (5)	
O10	0.67103 (19)	0.06452 (17)	0.37199 (17)	0.0590 (4)	
C11	0.6948 (3)	0.1230 (2)	0.2347 (2)	0.0499 (5)	
C12	0.7212 (3)	−0.0736 (2)	0.4230 (2)	0.0494 (5)	
C13	0.7864 (3)	−0.1617 (2)	0.3361 (2)	0.0468 (5)	
C14	0.7604 (3)	0.0446 (2)	0.1403 (2)	0.0466 (5)	
C15	0.7710 (4)	0.3410 (3)	−0.1775 (3)	0.0837 (8)	
H153	0.8406	0.4550	−0.1321	0.126*	
H152	0.6498	0.3312	−0.2760	0.126*	
H151	0.8401	0.2873	−0.2153	0.126*	

### Atomic displacement parameters (Å^2^)

**Table:**

	*U*^11^	*U*^22^	*U*^33^	*U*^12^	*U*^13^	*U*^23^
C1	0.0528 (13)	0.0580 (13)	0.0503 (12)	0.0096 (10)	0.0242 (10)	0.0068 (9)
C2	0.0544 (13)	0.0574 (13)	0.0561 (12)	0.0066 (10)	0.0185 (10)	0.0142 (10)
C3	0.0636 (15)	0.0503 (12)	0.0768 (15)	0.0177 (11)	0.0232 (12)	0.0157 (11)
C4	0.0650 (15)	0.0579 (14)	0.0772 (15)	0.0234 (11)	0.0338 (12)	0.0065 (11)
C5	0.0607 (14)	0.0784 (15)	0.0507 (12)	0.0146 (12)	0.0324 (11)	0.0058 (11)
C6	0.0706 (16)	0.0855 (17)	0.0549 (13)	0.0158 (13)	0.0320 (12)	0.0220 (12)
C7	0.0798 (17)	0.0697 (15)	0.0640 (13)	0.0251 (13)	0.0324 (12)	0.0262 (11)
C8	0.0670 (14)	0.0558 (13)	0.0586 (12)	0.0193 (11)	0.0302 (11)	0.0096 (10)
C9	0.0470 (11)	0.0518 (11)	0.0494 (11)	0.0120 (9)	0.0263 (9)	0.0043 (9)
O9	0.1003 (12)	0.0713 (10)	0.0769 (10)	0.0395 (9)	0.0617 (9)	0.0172 (8)
O10	0.0687 (10)	0.0625 (9)	0.0603 (9)	0.0252 (7)	0.0400 (7)	0.0096 (7)
C11	0.0468 (12)	0.0492 (11)	0.0510 (11)	0.0117 (9)	0.0225 (9)	0.0055 (9)
C12	0.0447 (11)	0.0525 (12)	0.0490 (11)	0.0103 (9)	0.0227 (9)	0.0054 (9)
C13	0.0432 (11)	0.0496 (11)	0.0441 (10)	0.0088 (9)	0.0205 (9)	0.0047 (8)
C14	0.0436 (11)	0.0463 (11)	0.0460 (10)	0.0076 (9)	0.0210 (9)	0.0045 (8)
C15	0.0831 (18)	0.0799 (17)	0.0763 (16)	0.0159 (14)	0.0302 (14)	0.0321 (13)

### Geometric parameters (Å, º)

**Table:**

C1—C2	1.373 (3)	C7—C8	1.373 (3)
C1—C14	1.400 (2)	C7—H7	0.9300
C1—H1	0.9300	C8—C13	1.402 (3)
C2—C3	1.396 (3)	C8—H8	0.9300
C2—C15	1.509 (3)	C9—O9	1.225 (2)
C3—C4	1.367 (3)	C9—C14	1.464 (3)
C3—H3	0.9300	C9—C13	1.467 (2)
C4—C11	1.385 (3)	O10—C12	1.368 (2)
C4—H4	0.9300	O10—C11	1.377 (2)
C5—C6	1.362 (3)	C11—C14	1.386 (3)
C5—C12	1.390 (3)	C12—C13	1.385 (3)
C5—H5	0.9300	C15—H153	0.9600
C6—C7	1.386 (3)	C15—H152	0.9600
C6—H6	0.9300	C15—H151	0.9600
			
C2—C1—C14	122.1 (2)	C13—C8—H8	119.6
C2—C1—H1	119.0	O9—C9—C14	122.70 (17)
C14—C1—H1	119.0	O9—C9—C13	122.40 (18)
C1—C2—C3	117.6 (2)	C14—C9—C13	114.91 (16)
C1—C2—C15	122.2 (2)	C12—O10—C11	118.91 (15)
C3—C2—C15	120.2 (2)	O10—C11—C4	116.28 (18)
C4—C3—C2	122.0 (2)	O10—C11—C14	122.97 (17)
C4—C3—H3	119.0	C4—C11—C14	120.75 (19)
C2—C3—H3	119.0	O10—C12—C13	122.48 (17)
C3—C4—C11	119.3 (2)	O10—C12—C5	116.03 (18)
C3—C4—H4	120.3	C13—C12—C5	121.50 (19)
C11—C4—H4	120.3	C12—C13—C8	117.83 (18)
C6—C5—C12	119.2 (2)	C12—C13—C9	120.58 (17)
C6—C5—H5	120.4	C8—C13—C9	121.59 (17)
C12—C5—H5	120.4	C11—C14—C1	118.27 (18)
C5—C6—C7	121.0 (2)	C11—C14—C9	119.92 (17)
C5—C6—H6	119.5	C1—C14—C9	121.80 (17)
C7—C6—H6	119.5	C2—C15—H153	109.5
C8—C7—C6	119.6 (2)	C2—C15—H152	109.5
C8—C7—H7	120.2	H153—C15—H152	109.5
C6—C7—H7	120.2	C2—C15—H151	109.5
C7—C8—C13	120.9 (2)	H153—C15—H151	109.5
C7—C8—H8	119.6	H152—C15—H151	109.5
			
C14—C1—C2—C3	−0.3 (3)	O10—C12—C13—C9	−0.4 (3)
C14—C1—C2—C15	179.37 (18)	C5—C12—C13—C9	179.75 (17)
C1—C2—C3—C4	0.7 (3)	C7—C8—C13—C12	0.3 (3)
C15—C2—C3—C4	−179.00 (19)	C7—C8—C13—C9	−179.92 (18)
C2—C3—C4—C11	0.0 (3)	O9—C9—C13—C12	−175.44 (19)
C12—C5—C6—C7	−0.2 (3)	C14—C9—C13—C12	4.3 (3)
C5—C6—C7—C8	0.1 (4)	O9—C9—C13—C8	4.8 (3)
C6—C7—C8—C13	−0.1 (3)	C14—C9—C13—C8	−175.48 (17)
C12—O10—C11—C4	−176.87 (17)	O10—C11—C14—C1	−178.18 (16)
C12—O10—C11—C14	2.7 (3)	C4—C11—C14—C1	1.3 (3)
C3—C4—C11—O10	178.55 (17)	O10—C11—C14—C9	1.6 (3)
C3—C4—C11—C14	−1.0 (3)	C4—C11—C14—C9	−178.92 (17)
C11—O10—C12—C13	−3.3 (3)	C2—C1—C14—C11	−0.7 (3)
C11—O10—C12—C5	176.62 (16)	C2—C1—C14—C9	179.57 (18)
C6—C5—C12—O10	−179.48 (18)	O9—C9—C14—C11	174.89 (19)
C6—C5—C12—C13	0.4 (3)	C13—C9—C14—C11	−4.9 (3)
O10—C12—C13—C8	179.45 (17)	O9—C9—C14—C1	−5.4 (3)
C5—C12—C13—C8	−0.4 (3)	C13—C9—C14—C1	174.86 (16)
